# A spatial transcriptomics comparison of the adult versus metamorphosed axolotl brain

**DOI:** 10.1038/s41597-026-06917-w

**Published:** 2026-02-24

**Authors:** Shuai Wang, Sulei Fu, Xiawei Liu, Yan-Yun Zeng, Lianrui Zhang, Nannan Zhang, Xi Dai, Hanbo Li, Ying Gu, Xiaoyu Wei, Ji-Feng Fei, Yang Liu

**Affiliations:** 1 College of Life Sciences, University of Chinese Academy of Sciences, Beijing, 101408 China; 2 State Key Laboratory of Genome and Multi-omics Technologies, BGI Research, Shenzhen, 518083 China; 3 Department of Pathology, Guangdong Provincial People’s Hospital (Guangdong Academy of Medical Sciences), Southern Medical University, Guangzhou, 510080 China; 4 BGI Research, Qingdao, 266555 China; 5 The Innovation Centre of Ministry of Education for Development and Diseases, School of Medicine, South China University of Technology, Guangzhou, 510006 China; 6 State Key Laboratory of Genome and Multi-omics Technologies, BGI Research, Hangzhou, 310030 China; 7 School of Basic Medical Sciences, Southern Medical University, Guangzhou, 510515 China

**Keywords:** Cellular neuroscience, Computational biology and bioinformatics

## Abstract

The Mexican axolotl (*Ambystoma mexicanum*) is an established model for studying tetrapod regeneration and development. Notably, axolotls exhibit remarkable brain regeneration as adults, a trait rarely observed in other adult vertebrates. Adult axolotls can undergo metamorphosis, a process that induces dramatic remodeling of multiple organs and is accompanied by a gradual decline in regenerative capacity and lifespan. However, systematic studies on whole-brain cellular dynamics and molecular mechanisms in both adult and metamorphosed individuals remain lacking. Here, we profiled five representative brain regions (olfactory bulb, telencephalon, diencephalon/mesencephalon, rhombencephalon, and pituitary) of the axolotl brain via spatial transcriptomics in both adult and metamorphosed individuals. Our work reveals metamorphosis-associated changes in cell types and molecular profiles across brain regions.

## Background & Summary

The Mexican axolotl (*Ambystoma mexicanum*) is an exceptional model organism renowned for its ability to regenerate complex structures, including limbs, tail, eyes, heart, lungs, gills, and central nervous system—a capability rarely observed in mammals^1–5^. This remarkable regenerative capacity positions the axolotl as a premier model for studying organ development and regeneration. A defining characteristic of axolotls is neoteny, the retention of larval features such as external gills and fins upon sexual maturity. They also exhibit resistance to age-related pathologies, contributing to an extended lifespan that is linked to their regenerative abilities^6^. Notably, exposure to specific environmental cues (e.g., reduced water levels) or thyroid hormone (T4) can induce metamorphosis in adults, triggering an irreversible transition to a terrestrial form characterized by the loss of aquatic larval features^6^. Crucially, this metamorphosis is accompanied by a significant decline in regenerative capacity. Previous studies have primarily examined the endocrine axis underlying metamorphosis^7–9^. Understanding the mechanisms that govern this process and its associated loss of regeneration is fundamental to elucidating the principles of regenerative control, with potential implications for manipulating regenerative potential in other organisms, including mammals. Consequently, deciphering the axolotl’s regenerative mechanisms offers valuable insights for advancing the field of regenerative medicine.

Recent advances in the assembly of the large axolotl genome and tissue-mapped *de novo* transcriptomics have paved the way for detailed studies of gene expression patterns^10,11^. The application of single-cell RNA sequencing (scRNA-seq) and spatial transcriptomics has enabled the investigation of brain regeneration following mechanical injury in axolotls^12,13^. By analyzing gene expression at single-cell resolution, these studies have identified injury-induced cell types and elucidated their roles in neural circuit regeneration and remodeling. A key finding was the discovery of distinct subpopulations of ependymoglial cells (EGCs) that are specifically induced during brain regeneration. In parallel, another study has used scRNA-seq to profile transcriptional changes during thyroxine-induced metamorphosis^14^.Given the strong link between metamorphosis and the decline in regenerative capacity and longevity, characterizing the accompanying molecular and cellular changes in the brain represents a critical research goal.

We applied SpaTial Enhanced REsolution Omics-sequencing (Stereo-seq^15^) to determine the spatially resolved single-cell transcriptomes of whole-brain sections from both Adult and Metamorphosed (Meta.) axolotls. Using these data, we identified 24 cell types, including EGC subtypes associated with metamorphosis. Following cell segmentation and a rigorous quality control pipeline, we obtained transcriptomic data from 19 spatial sections, yielding a total of 83,054 high-quality, spatially resolved single-cells. Integration of spatial information with gene expression profiles revealed spatial heterogeneity and specific molecular signatures within the metamorphosing brain. The single-cell resolution spatial transcriptome data resource generated in this study facilitates the understanding of molecular regulatory networks underlying amphibian metamorphosis and provides a valuable resource for regenerative medicine research.

## Methods

### Animal care and ethics

Animals used in this study all originated from the Ambystoma Genetic Stock Center collection (USA), maintained and bred in the laboratory of Dr. Ji Feng Fei at Guangdong Provincial People’s Hospital. Animals were housed in dechlorinated tap water at 18–20 °C under a 12 h light/12 h dark cycle, with daily feeding. Adult male animals (non metamorphosed adults) selected in this study were 20 months of age with a body length of 21–24 cm. For induced metamorphosis, adult male axolotls (12 months of age) received an intraperitoneal injection of L thyroxine (T4; 1.5 mg/kg), and exhibited complete metamorphic features within 30 days post-injection. Tissue collection was performed after 6 months post-induction (at 18 months of age for metamorphosed adults with a body length of 21–24 cm). While a two-month age difference exists between groups, this interval is negligible relative to the axolotl lifespan (10–15 years), and both groups represent sexually mature adults with similar body sizes, minimizing potential aging-related confounding factors prior to the onset of significant senescence.

For brain tissue collection, animals were deeply anesthetized in 0.03%(w/v) benzocaine (Sigma, E1501-500G) until loss of righting reflex and absence of response to tail/foot pinch were confirmed, then euthanized by a secondary physical method (cervical transection) to ensure death prior to dissection. Animal health and welfare were monitored daily. All·animal procedures were conducted in compliance with Chinese animal·welfare legislation and institutional standard operating procedures, and were approved by the Biomedical Research Ethics Committee of Guangdong Provincial. People’s hospital (KY-Q-2022-395-01).

### Tissue collection and cryosection

The entire brains were rapidly collected from the cranial cavity of euthanized animals by opening the skull, with all procedures performed on ice. The collected brain samples were freshly embedded in Tissue-Tek® OCT Compound (4583, Sakura, Torrance, CA) and then rapidly frozen at −80°C. Subsequently, cryosectioning was performed sequentially along the anterior-posterior axis across different brain regions to generate 20 μm-thick frozen sections. During the sectioning process, each tissue section was examined microscopically to ensure morphological comparability of brain sections between adult and metamorphic animals.

### Spatial transcriptomics sequencing via Stereo-seq

Tissue sections were mounted on a pre-washed Stereo-seq capture chip, fixed in methanol, and fluorescently stained for imaging using a Motic microscope. After permeabilization with pepsin/HCl, captured RNAs were reverse-transcribed on-chip with SuperScript II reverse transcriptase and a template-switching oligonucleotide (TSO). cDNA was amplified via PCR using KAPA HiFi HotStart ReadyMix, purified, and fragmented with Tn5 transposase. Libraries were constructed by PCR with Stereo-seq-specific primers, purified with clean beads, and sequenced on an MGI DNBSEQ-T1 instrument (35 bp read1, 100 bp read2).

### Stereo-seq raw data processing

Raw sequencing data were generated using an MGI DNBSEQ-T1 sequencer. Reads were aligned to the reference genome AmexG_v6.0 using STAR (v2.7.6a)^16^. Mapped reads with MAPQ > 10 were processed with *handleBam* to annotate transcriptional origins: reads with >50% exon overlap were classified as exonic; those with <50% exon overlap but overlapping adjacent introns were considered intronic; otherwise, they were labeled intergenic. UMIs with identical CIDs and gene loci were collapsed, allowing one mismatch for PCR error correction. A CID-based expression matrix was finally generated using exonic reads.

### Single-cell identification in stereo-seq data via circling method

Cell regions were identified based on ssDNA staining images aligned with Stereo-seq grayscale DNB maps, following the method described by Wei *et al*.^13^. After manual registration, an automatic OTSU algorithm was applied for global thresholding and background removal, followed by a Gaussian-weighted local threshold with a block size of 41 and an offset of 0.03 was applied to detect cell locations. Overlapping cells were separated using Euclidean distance transformation and marker-based watershed segmentation with a minimal distance of 15 via scikit-image (v0.24.0)^17^. Cell originating from non-target tissues were excluded.

### Quality control, integration, and annotation

The obtained data underwent quality control by filtering out cells with fewer than 200 UMIs. Normalization was performed using *SCTransform* from Seurat (v4.1.0), followed by integration across samples with Canonical Correlation Analysis (CCA) to mitigate potential batch effects^18^. The integrated dataset was visualized via uniform manifold approximation and projection (UMAP) dimensionality reduction and annotated into cell types based on canonical marker genes.

### Differential gene expression analysis

Differentially expressed genes (DEGs) were identified across all cell types between Adult and Meta. stages using *FindMarkers* (Wilcoxon rank-sum test; |avg_logFC| > 0.25, adjusted p-value < 0.05).

### Spatial autocorrelation analysis (Moran’s I)

To assess spatial clustering of gene expression, we computed global Moran’s I indices using a custom Python implementation. The analysis was performed on 2D cell coordinates and gene expression vectors, applying inverse-distance weighting (*power* = 2.0) to construct the spatial weight matrix, which was row-standardized. A Moran’s I > 0 indicates positive spatial autocorrelation, while I < 0 reflects spatial dispersion.

### Proportional analysis

Cell type proportion differences were assessed with a hypergeometric test. To mitigate sampling bias, down-sampling was performed within the same brain regions across stages.

### Cell communication analysis

Cell-cell communication was analyzed per spatial slice using CellChat (v2.1.0)^19^. Ligand–receptor interactions were inferred based on *CellChatDB.human*, with axolotl genes converted to human homologs. Communication probabilities were computed with *computeCommunProb* using spatial constraints (*distance.use* = TRUE, *interaction.range* = 250, *scale.distance* = 3). Interactions involving fewer than 10 cells were filtered out via *filterCommunication* and set to NA.

## Data Records

We generated Stereo-seq spatial transcriptomes from five representative brain regions of both Adult and Meta. axolotls (Table 1). This dataset, comprising 19 sections, surveys the major regions of the adult axolotl brain.

**Table 1:** Sample information and quality metrics.

No.	Tissue	Stage	Animal ID	Sex	Age (months)	Mean genes per cell	Mean UMIs per cell	Mean spots per cell	Cell number
1	OB	Adult	A	Male	20	1069.9	3415.1	759.3	6659
2	OB	Adult	A	Male	20	1374.5	4559.9	802.7	6261
3	Tel.	Adult	A	Male	20	1547.5	5085.4	737.8	7987
4	Tel.	Adult	A	Male	20	1082.4	3559.3	753.2	7566
5	Dien./Mes.	Adult	A	Male	20	1873.8	7383.6	1055.9	4530
6	Dien./Mes.	Adult	A	Male	20	1560.1	5805.2	768.2	5837
7	Dien./Mes.	Adult	A	Male	20	1508.5	5575.3	778.3	5237
8	Rho.	Adult	A	Male	20	1384.5	5034.8	751.3	3188
9	Rho.	Adult	A	Male	20	939.7	3253.8	702.3	1296
10	Pituitary	Adult	A	Male	20	1682.8	8163.6	1007.6	1364
11	OB	Meta.	B	Male	18	1187.2	3257.7	891.5	5370
12	OB	Meta.	B	Male	18	1945.6	6581.1	748.4	5989
13	Tel.	Meta.	B	Male	18	1216.8	2999.2	793.5	6669
14	Dien./Mes.	Meta.	B	Male	18	1707.2	5599.6	725.7	3013
15	Dien./Mes.	Meta.	B	Male	18	981.0	2526.5	773.8	3537
16	Dien./Mes.	Meta.	B	Male	18	1085.5	3174.7	853.4	2524
17	Rho.	Meta.	B	Male	18	1336.2	4814.7	846.5	3219
18	Rho.	Meta.	B	Male	18	739.4	2291.5	681.1	1167
19	Pituitary	Meta.	B	Male	18	930.8	3698.1	704.0	1641

Raw sequencing data, including 72 FASTQ files, are stored in the CNGB Nucleotide Sequence Archive (CNP007968^20^) and NCBI SRA (split into 173 smaller FASTQ files, SRP666329^21^).

Processed data, including 19 AnnData H5AD files (one for each slice, including segmented cell bin matrix, cell coordinates and annotation metadata.), 19 GEM files (spot-level spatial gene expression matrices in long-format), and 19 ssDNA images (preprocessed and aligned ssDNA images) are publicly available on Figshare (10.6084/m9.figshare.30082183)^22^ and NCBI GEO (GSE317755^23^).

Figshare^22^ also houses all resources necessary to reproduce the analyses and supplemental results. This includes: *ProcessedData.tar.gz*, containing 19 H5AD files and an integrated RDS file; *Code.tar.gz*, containing all pipeline analysis and technical validation code; *GEM.tar.gz*, storing the spot-level spatial gene expression matrices in long-format (19 files); *ProcessedSsDNA.tar.gz*, storing the preprocessed and aligned ssDNA images (19 files); *SpatialFeaturePlot.tar.gz* stores the spatial feature plots of marker genes and DGE genes mentioned in this work (74 files); *SpatialUMIPlot.tar.gz* stores the spatial distribution plots of UMI counts (19 files); additionally, *Result.tar.gz* stores the metadata, annotation information, marker lists, and Moran’s I results for technical validation (6 files).

*SampleRecord.xlsx* on Figshare^22^ records in detail the CNGB Sample, NCBI BioSample, SRA Run, and GEO Series for each slice, along with their corresponding processed filenames.

## Technical Validation

**Fig. 1:**
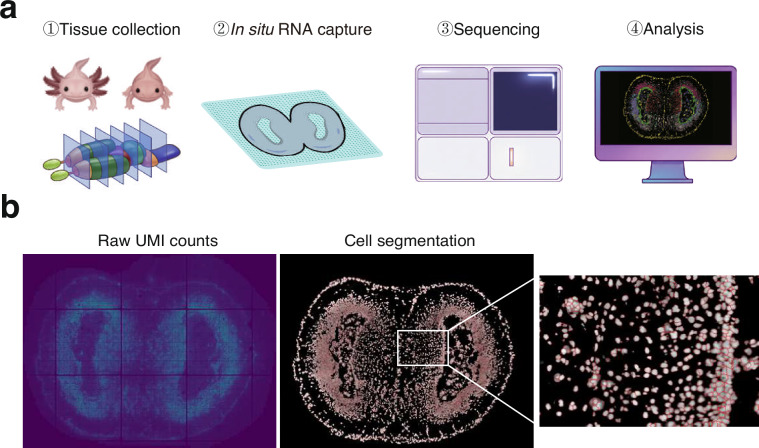
Stereo-seq workflow of axolotl brain. (**a**) Schematic diagram of the experimental and computational workflow. 19 cryosections from five brain regions of Adult and Meta. axolotls underwent Stereo-seq spatial transcriptomics sequencing, followed by downstream bioinformatics analysis for data processing and interpretation. (**b**) Representative spatial distribution of raw UMI counts per spot (left) and cell segmentation boundaries (red outlines) overlaid on the corresponding ssDNA staining image, with a magnified view (right).

In this study, 19 cryosections from various brain regions of both Adult and Meta. axolotls were subjected to spatial transcriptomic sequencing (Fig. 1a). We first evaluated the performance of cell segmentation. Representative spatial distribution of sequencing depth (raw UMI counts per spot) shows that the total UMI counts at each spot aligned with tissue morphology, with low signal in background regions and no apparent spatial bias or abrupt loss of signal indicative of technical artifact (Fig. 1b left). Cell segmentation boundaries and corresponding ssDNA staining images are shown, confirming that the segmentation method robustly identified cells based on nuclear staining in axolotl brain tissues (Fig. 1b right). The complete sets of spatial UMI distribution maps and ssDNA staining images for all samples are available on Figshare^22^.

**Fig. 2:**
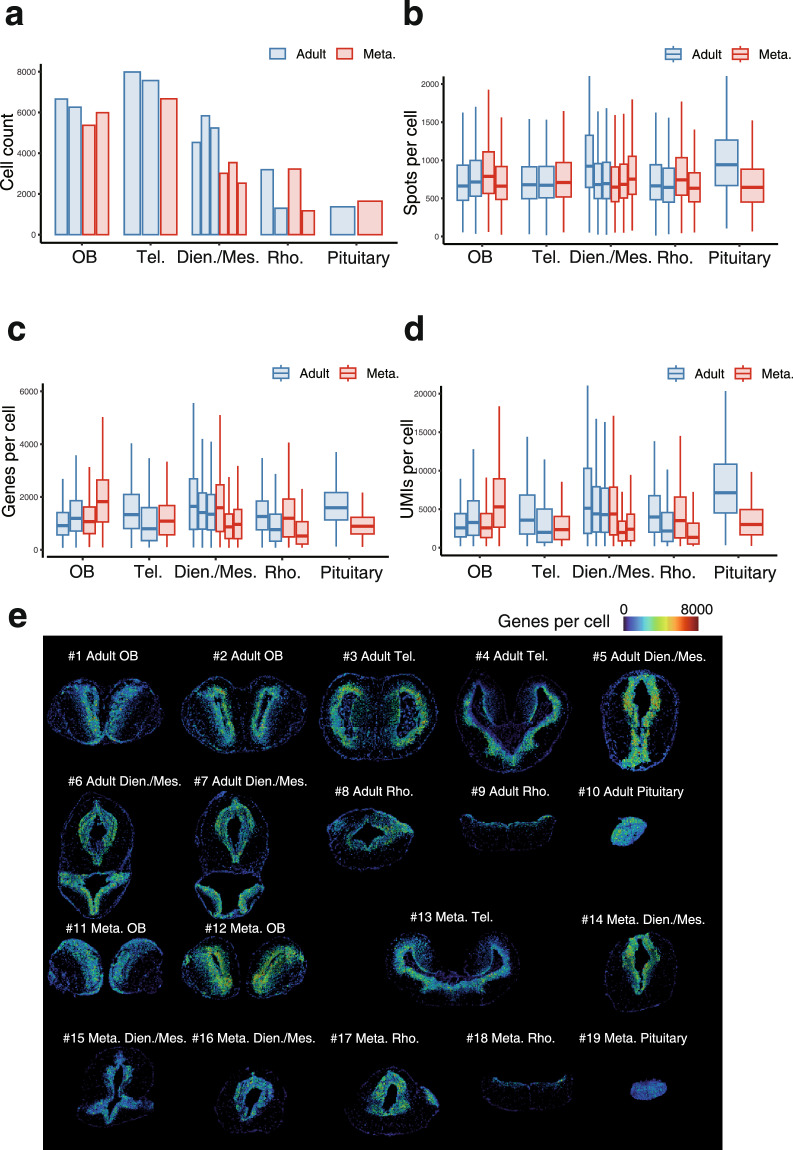
Quality control of Stereo-seq slices. (**a**) Bar graph showing the total cell counts in each slice from adult (blue) and Meta. (red) axolotls. (**b**–**d**) Distribution of spots, genes and unique molecular identifiers (UMIs) per cell in each slice from Adult (blue) and Meta. (red) axolotls. (**e**) Spatial distribution of genes per cell across all 19 slices.

After filtering, a total of 83,054 high-quality cells were retained, with averages of 794 DNA nanoballs (DNBs), 1,371.3 genes, and 4,596.3 UMIs detected per cell (Table 1, and Fig. 2a–d). The uniformity of gene capture efficiency, both within and between slices, was demonstrated by spatial visualization of the number of detected genes per cell (Fig. 2e). On average, 84.17 ± 10.00% (mean ± SD) of transcripts were located within the segmented cells, indicating good tissue coverage. Furthermore, the average Moran’s I for the top 100 highly variable genes across all slices was 0.11 ± 0.04 (mean ± SD), demonstrating favorable spatial gene structure (the results are available on Figshare^22^).

**Fig. 3:**
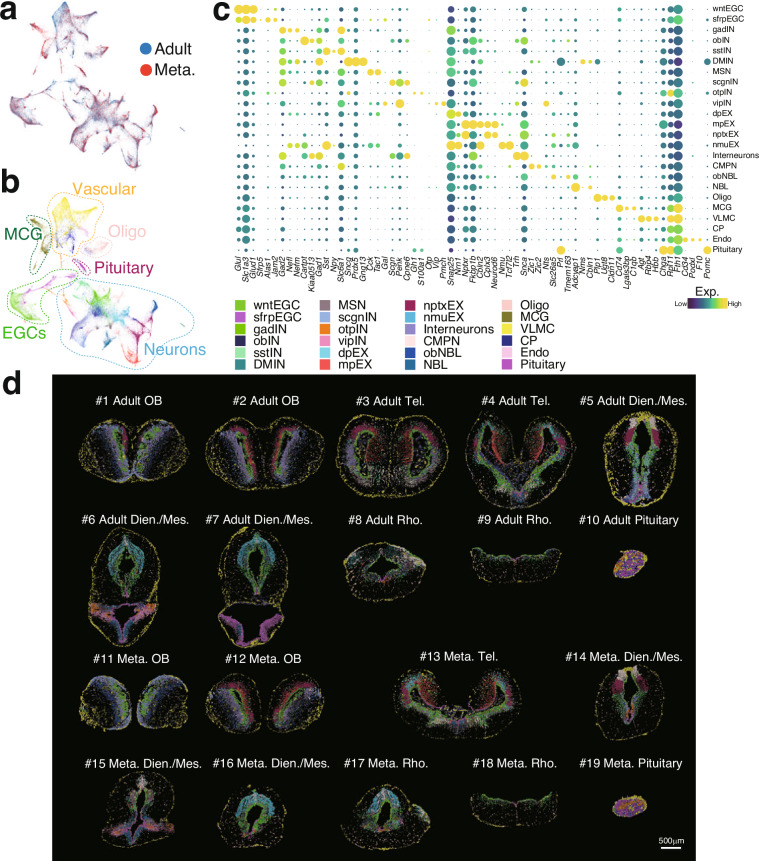
Identification of cell type in Adult and Meta. axolotl brain. (**a,b**) UMAP visualization of integrated cells in Adult and Meta. brain, colored by group identity and cell type. (**c**) Dot plot showing top marker genes for each identified cell type. Color represents average scaled expression level, and dot size indicates the percentage of cells expressing the marker. (**d**) Spatial distribution of identified cell types across all 19 slices.

**Fig. 4:**
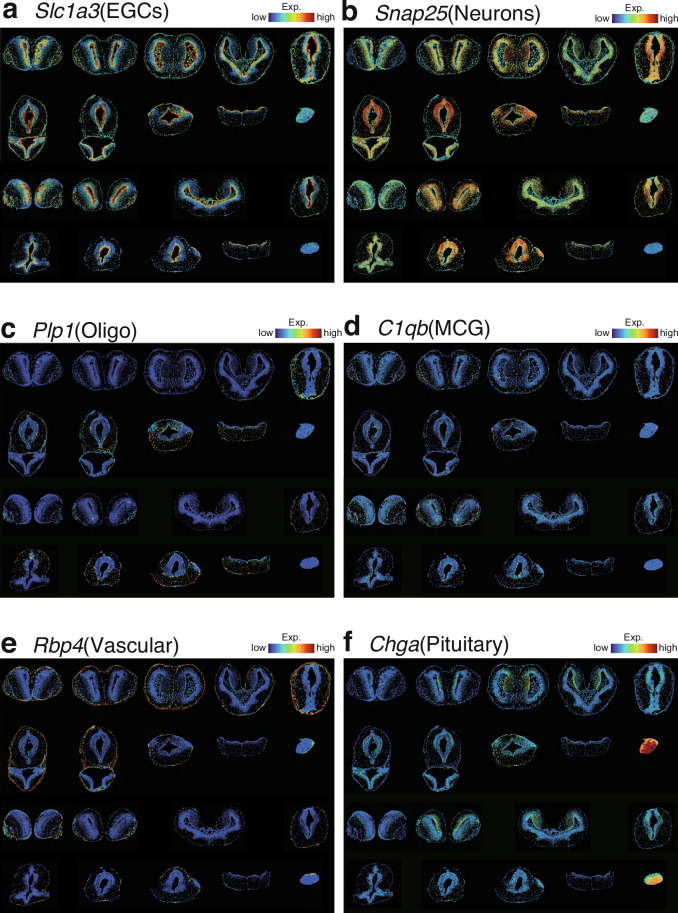
Spatial expression patterns of key cell type marker genes. Representative spatial feature plots of *Slc1a3* (**a**), *Snap25* (**b**), *Plp1* (**c**), *C1qb* (**d**), *Rbp4* (**e**), *Chga* (**f**).

To evaluate data reproducibility and batch effects between Adult and Meta. samples, we applied CCA to assess integration performance. Overlapping of cell clusters in the manifold space indicates that technical batch effects were effectively removed while biological heterogeneity was preserved (Fig. 3a). Through differential gene expression analysis, we identified 24 distinct cell types. These include two types of EGCs: venEGC(ventricle EGC) and infEGC (infundibulum EGC), six subtypes of inhibitory neurons, such as obIN (olfactory bulb inhibitory neuron), gadIN, sstIN, scgnIN, otpIN, vipIN (marked by *Gad*^+^, *Sst*^+^, *Scgn*^+^, *Otp*^+^, and *Vip*^+^ expression, respectively), and DMIN (dopaminergic periglomerular inhibitory neuron); four types of excitatory neurons: dpEX and mpEX (dorsal and medial pallium excitatory neurons), nptxEX, and nmuEX; three other neuronal classes: MSN (medium spiny neuron), interneurons, and CMPN (cholinergic, monoaminergic, and peptidergic neuron); two neuroblast types, NBL and obNBL (olfactory bulb neuroblast); as well as Oligo (oligodendrocyte), MCG (microglial cell), VLMC (vascular leptomeningeal cell), CP (choroid plexus), Endo (endothelial cell), and Pituitary (pituitary cell) (Fig. 3b–d). Marker list are available on Figshare^22^. Key marker genes used for annotation were validated by their spatial expression patterns (Fig. 4 and a complete set on Figshare^22^).

**Fig. 5:**
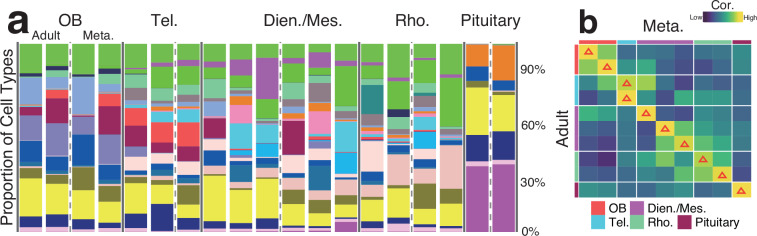
Comparative analysis of cell type composition between Adult and Meta. axolotl brain. (**a**) Stacked bar plot showing the proportional abundance of cell types in each slice. (**b**) Heatmap displaying transcriptomic correlation between corresponding slices from Adult and Meta. groups (red triangle indicates the slice pair with the highest correlation).

The spatial distribution and proportional composition of cell types were highly consistent between Adult and Meta. Samples (Fig. 3d and Fig. 5a). High transcriptomic similarity (Pearson correlation r = 0.71 ± 0.23) in corresponding brain regions, with the highest correlation uniquely observed between corresponding pairs, further confirms the robustness and reliability of the dataset (Fig. 5b).

**Fig. 6:**
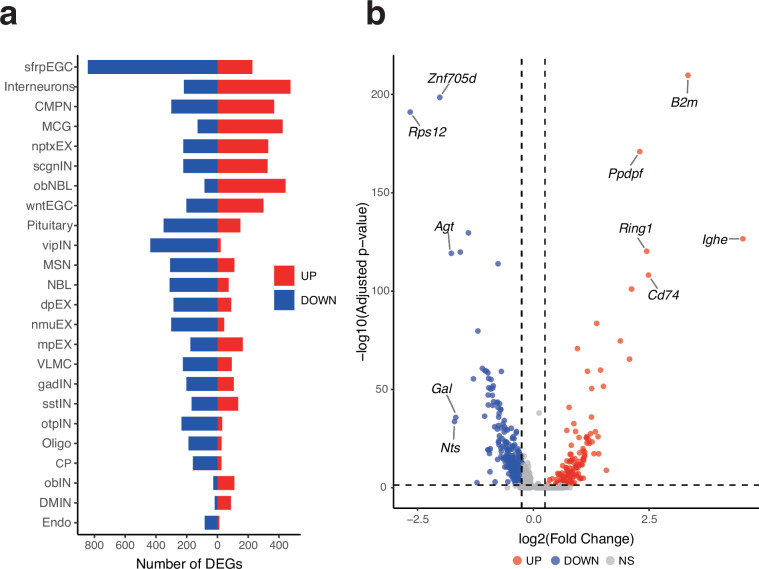
Identification of differentially expressed genes (DEGs) in Adult versus Meta. axolotl brains. (**a**) Number of DEGs per cell type in Meta. compared to Adult; UP (red) indicates upregulated genes, DOWN (blue) indicates downregulated genes. (**b**) Volcano plot of DEGs in infEGC from Meta. relative to Adult, highlighting top DEGs. NS, not significant.

To validate that the dataset captures biologically relevant molecular changes, we performed differential gene expression analysis. Results revealed that specific cell types such as EGCs, particularly the infEGC subtype, exhibited the highest number of DEGs (1,113 in total) (Fig. 6a). The detection of expected metamorphosis-related markers, including the upregulation of *Ighe*, *B2m*, *Cd74*, *Ring1*, and the downregulation of *Rps12*, *Znf705d*, *Agt*, confirms the data’s sensitivity to biological state changes (Fig. 6b). DEG list and spatial plots are available on Figshare^22^.

**Fig. 7:**
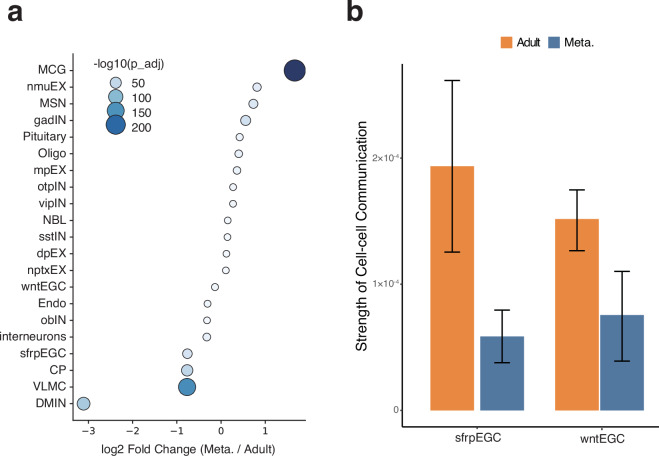
Changes in cell proportion and cell-cell communication between Adult and Meta. axolotl brains. (**a**) Bubble plot showing proportional changes of major cell types in Meta. compared to Adult; color and size of bubbles indicate the −log_10_(adjusted p-value). (**b**) Bar graph comparing the strength of cell-cell communication between microglial cells (MCGs) and ependymoglial cells (EGCs) in Adult and Meta. Groups. Error bars represent the standard error of the mean (SEM).

Finally, we validated the dataset’s ability to infer cell-cell interactions. The analysis indicated a moderate decrease in EGCs and a pronounced increase in MCG proportions (Fig. 7a). Cell-cell communication analysis captured enhanced interaction strength between MCG and EGCs following metamorphosis (Fig. 7b), verifying that the dataset retains sufficient depth to resolve intercellular signaling networks.

In summary, this study established a spatially resolved transcriptomic survey of the whole brain in both adult and metamorphosed axolotls. This work provides a resource for further investigation into cellular dynamics during brain development and regeneration in *Ambystoma mexicanum*.

## Usage Notes

All analyses were performed in a Linux environment using R (v4.2.2) and Python (v3.8.14). Raw sequencing reads were aligned to the axolotl genome (AmexG_v6.0) with STAR (v2.7.6a). Cell segmentation was conducted via custom scripts using scikit-image (v0.24.0). Data integration, normalization, and clustering were performed with Seurat (v4.1.0). Spatial visualizations and Moran’s I scores were generated using custom Python scripts. Cell-cell communication was inferred via CellChat (v2.1.0). All processed data and code are available on Figshare^22^ to ensure reproducibility.

## Data Availability

The dataset is available at the CNSA (10.26036/CNP0007968), NCBI BioProject (https://identifiers.org/ncbi/bioproject/PRJNA1397965), NCBI SRA (https://identifiers.org/ncbi/insdc.sra:SRP666329), NCBI GEO (https://identifiers.org/geo/GSE317755) and Figshare (10.6084/m9.figshare.30082183).

## Code availability

The source code for downstream analysis and visualization is publicly available on Figshare (10.6084/m9.figshare.30082183).
